# Screening and Surveillance Bias in Cancer

**DOI:** 10.3390/epidemiologia4020012

**Published:** 2023-03-24

**Authors:** Stefano Tancredi, Stéphane Cullati, Arnaud Chiolero

**Affiliations:** 1 Population Health Laboratory (#PopHealthLab), University of Fribourg, 1700 Fribourg, Switzerland; 2 Department of Readaptation and Geriatrics, University of Geneva, 1211 Geneva, Switzerland; 3 School of Population and Global Health, McGill University, Montréal, QC H3A 1G1, Canada

**Keywords:** surveillance bias, cancer, cancer risk factors, cancer surveillance

## Abstract

Surveillance bias arises when differences in the frequency of a condition are due to changes in the modality of detection rather than to a difference in the actual risk of the condition. This bias hampers the surveillance of scrutiny-dependent cancers, leading to misinterpretations of cancer trends, risk factor identification, and, consequently, to the wrong public health actions.

## 1. Surveillance Bias of Cancer

What is the true burden of cancer? According to data from the International Agency for Research on Cancer (IARC), 10 million people worldwide died of cancer and 19 million new cases arose in 2020 [1]. It is standard to use mortality, incidence, and survival to assess the burden of cancer and the progress made in cancer control. However, for some types of cancers, specifically the so-called “scrutiny-dependent cancers” (Table 1) [2], using incidence or survival rates to assess the burden of cancer can be problematic because these metrics are influenced by the modality of detection of these cancers. Therefore, in settings of intense screening activities, the incidence and survival rates can be relatively high compared to settings without this level of screening activities, whatever the benefits of screening. If these rates are taken for granted, this results in “surveillance bias”, a type of bias that occurs when differences across time, settings, or populations in the detection activities of scrutiny-dependent cancers lead to differences in the incidence, which are wrongly attributed to changes in the actual risk of these cancers [3].

To understand what surveillance bias is, it helps to consider how it relates to the problem of overdiagnosis. In oncology, as in other fields of medicine, the ever-evolving testing and imaging techniques allow us to find anomalies previously not detected. On the one hand, this opens the way to great opportunities for new early detection and screening strategies for cancer. On the other hand, because these anomalies are not all associated with a substantial health hazard, there is no benefit to being aware of them; moreover, if these harmless anomalies are diagnosed as diseases, this is a situation of overdiagnosis [4]. More broadly, this is linked to the well-known “length-time bias” associated with all types of early detection and screening activities. Much less known is the major impact of this phenomenon on the surveillance of some types of cancer because it biases incidence trends due to the confusion between the diagnosis of harmless anomalies and the diagnosis of clinically relevant disease [5].

**Table 1:** Surveillance bias in scrutiny-dependent cancers.

Type of Cancer	Comment
Thyroid cancer	Thyroid cancer is particularly sensitive to the intensity of screening and clinical detection activities due to a large reservoir of indolent cancer subtypes, such as small papillary thyroid cancers [6,7].
Prostate cancer	The incidence of prostate cancer changed in many countries following changes in the proportion of people having PSA-based screening [8,9].
Melanoma	Routine screening is not recommended, but melanoma incidence is influenced by the frequency of skin checks [10].
Breast cancer	Breast cancer screening leads to some cases of overdiagnosis, influencing the analysis of incidence trends [11].
Kidney cancer	The incidence of kidney cancer is influenced by incidental detection due to increased utilization of imaging techniques [12].

## 2. Screening as a Cause of Cancer

Screening has a major influence on the incidence of some types of cancer. There are several examples of how surveillance bias due to screening activities hinders the analysis of cancer trends. For instance, in the United States, the incidence of prostate cancer sharply increased at the beginning of the 1990s following the introduction of prostate-specific, antigen-based screening; however, following the 2012 USPSTF recommendation against routine screening, the incidence has decreased (predominantly for early stage and low-grade cancers) [8,9]. However, these changes in incidence were not associated with concomitant changes in mortality. If surveillance was based only on the analysis of incidence, one could suspect that these trends are due to increased exposure to carcinogenic agents or unhealthy behaviors, yet they result from changes in early detection and screening activities over time.

Melanoma is also at risk of surveillance bias [6]. As for prostate cancer, mortality is less likely to bias the assessing of the true burden of this cancer. While melanoma incidence rates have risen in many countries, mortality has either remained stable or decreased in the past few years, likely due to new treatments for metastatic disease. This decoupling between incidence and mortality rates can be the sign of overdiagnosis. Recent estimates showed that roughly 60% of melanoma cases could be overdiagnosed in the United States, and similar proportions of overdiagnosis were reported in Australia [13,14]. Although routine screening is not recommended in most countries, melanoma incidence is influenced by the intensity of skin checks [10,15]. Due to differences in skin checks, the huge differences in incidence across countries do not reflect true differences in the risk of clinically meaningful melanoma—that is, cases that require treatment and that we aim to prevent.

## 3. Bias in Risk Factor Identification

Surveillance bias can also lead to a misinterpretation of potential risk factors for the occurrence of cancer since screening uptake, screening availability, access to health services, and care-seeking behaviors differ between population subgroups. A higher frequency of medical examinations in specific population groups can indeed lead to a higher probability of detecting scrutiny-dependent cancers, and this higher probability can be misinterpreted as higher disease risk [2]. For example, a higher risk of cancer in obese people could be due, on the one hand, to a genuine effect of obesity on cancer risk, or, on the other hand, the higher risk could be due to the fact that obesity is associated with more frequent medical inquiries and hence a greater probability of finding cancers [16]. Another example is the higher incidence of thyroid cancer in women compared to men. While one explanation is that higher estrogen levels in women may increase the risk of developing thyroid cancer, this difference in incidence may only be apparent [17] and could be the result of differences in care-seeking behavior or physicians’ clinical practice. Actually, women may be more likely to have their thyroids checked, and physicians may be more likely to investigate thyroid cancer in women.

A final example is the increased rates of breast cancer in women with a high socioeconomic status, which could be due to a higher screening uptake rather than a genuinely higher risk of breast cancer in women with a high socioeconomic status [2].

## 4. Prevention of Surveillance Bias

There are several strategies to cope with surveillance bias (Table 2). When looking at cancer trends, it is important to account for differences in screening and diagnostic strategies over time and to focus on indicators that are less susceptible to surveillance bias. Accounting for this bias is particularly important for the communication of information about cancer to the population because people are highly fearful of cancer and easily misinterpret changes in incidence. Moreover, we should also be aware that assessing the risk factors, such as socioeconomic determinants linked to health care use, for scrutiny-dependent cancers based on incidence data can be misleading.

In conclusion, surveillance bias can influence cancer trends and the identification of cancer risk factors, hindering surveillance activities and potentially leading to the wrong public health actions. Being aware of this bias allows for a better assessment of the true evolution of the burden of cancer.

## Figures and Tables

**Table 2:** Four strategies to prevent surveillance bias in cancer burden assessment.

1) Analyze trends accounting for screening and diagnostic processes.
2) Focus on indicators that are less susceptible to surveillance bias, such as mortality trends or incidence trends of advanced cancer (stages 3 and 4).
3) Prefer data specifically designed for surveillance purposes (e.g., data from cancer registries).
4) Standardize the definition of the condition across surveillance systems and across time.

## Data Availability

Not applicable.
